# Widespread 25-Hydroxyvitamin D Deficiency in Affluent and Nonaffluent Pregnant Indian Women

**DOI:** 10.1155/2014/892162

**Published:** 2014-06-18

**Authors:** Rati Jani, Suhaila Palekar, Tanya Munipally, Padmini Ghugre, Shobha Udipi

**Affiliations:** Department of Food Science and Nutrition, SNDT Women's University, Juhu Tara Road, Santacruz (West), Mumbai, Maharashtra 400049, India

## Abstract

*Objectives*. This cross-sectional study primarily aimed to assess vitamin D adequacy in the third trimester of pregnancy using 25-hydroxyvitamin D (25(OH)D) and explore lifestyle characteristics (sun exposure index, diet, and economic indicators) associated with serum 25(OH)D. The secondary aim was to examine the relationship of serum 25(OH)D with birth weight and gestational age. *Methods*. Serum 25(OH)D was measured by chemiluminescent immunoassay in 150 pregnant women from Mumbai. Sun exposure index was computed. Dietary calcium, phytate : calcium ratio, and dietary phosphorus was calculated using the 24-hour diet recall method. *Results*. All women had 25(OH)D levels < 30.00 ng/ml. Multivariable linear regression showed that nonaffluent women had poorer 25(OH)D status than their affluent counterparts (*β* = −0.20; *P* = 0.03). Higher sun exposure index was associated with higher 25(OH)D concentrations (*β* = 0.31; *P* < 0.001), which remained significant after controlling for covariates. At the bivariate level, mothers of infants weighing <2500 g had lower serum 25(OH)D concentrations compared to mothers whose infants weighed ≥2500 g (*P* = 0.02). This association became non-significant after controlling for covariates. *Conclusions*. Vitamin D deficiency was universally prevalent in the cohort studied. There is a need to develop culturally sensitive strategies for improving the 25(OH)D status.

## 1. Introduction

India is close enough to the equator (latitude: 22° 00′ N; longitude: 77° 00′ E) to receive sufficient ultraviolet B radiations (wavelength: 290 to 315 nm) year-round and therefore should not experience poor vitamin D status. However, evidence indicates otherwise [1]. Widespread suboptimal (<30 ng/mL) status of 25(OH)D has been reported among diverse groups which include adults [2], pregnant women [3], postmenopausal women [4], children, and adolescents [5] belonging to both urban and rural [2, 6] and affluent and nonaffluent sectors [5], with circulating 25(OH)D levels averaging between 34.8 and 46.3 nmol/L. This paradox could be explained by several factors such as modest clothing (e.g.,* sarees* worn by women) which only allows the feet and arms to be exposed to sunshine, urbanization and social norms limiting outdoor activities for women, and pollution in cities. Further, the naturally dark skin pigmentation may reduce the synthesis of vitamin D [7].

Approximately 90% of the vitamin D requirements are met via UV B radiations, which penetrate the skin and convert 7-dehydrocholesterol to 25-hydroxyvitamin D, which is further converted to 1,25-dihydroxyvitamin D [8]. Most of the vitamin D in the human body is derived from exposure to sunlight, whereas contribution from dietary sources is limited [8]. In India, the contribution of dietary sources is poor because foods are not fortified with vitamin D, religious beliefs, that is, vegetarianism, prohibit intake of fish, and financial constraint limits regular consumption of these foods [9].

The extensively acknowledged function of vitamin D is to regulate calcium and phosphate metabolism. Calcium plays an important role in bone mineralization, muscle contraction, functioning of the nervous system, and cellular activities [10]. The importance of meeting vitamin D requirements and maintaining adequate intake through the life cycle cannot be emphasized enough and pregnancy unarguably is one of the critical stages during which nutritional requirements are increased [8]. Breast milk is a relatively poor source of vitamin D; therefore, in order to maintain an optimal vitamin D status during early infancy, it is essential for the mother to have an adequate vitamin D status during pregnancy [11]. This is supported by evidence which highlights that 25(OH)D readily crosses the placental membrane; thus, the fetal vitamin D pool is entirely dependent on that of the mother [11]. Therefore, vitamin D inadequacy in pregnancy may lead to significant morbidity in both the mother and fetus. Hypovitaminosis D during pregnancy has been associated with an increased risk of preeclampsia, gestational diabetes mellitus, preterm birth, small-for-gestational-age infants, neonatal hypocalcaemia, tetany, and infantile rickets which heightens the risk of lower respiratory tract infections, a significant cause of infant mortality [1].

This study primarily aimed to examine vitamin D adequacy in the third trimester of pregnancy using serum 25(OH)D and explore lifestyle characteristics (sun exposure index, diet, and economic indicators) associated with serum 25(OH)D levels. The secondary aim was to study the relationship of serum 25(OH)D with birth weight and gestational age.

## 2. Methods

### 2.1. Selection of Subjects

This cross-sectional study was conducted between February and May 2013 (spring–summer), after receiving Ethical Approval from the Independent Ethics Committee, Mumbai. Several private and public medical centers across Mumbai were approached. Only two medical centers, a private, fee-paying nursing home (affluent) and a non-fee-paying hospital (nonaffluent), gave permission for recruitment. The two medical facilities are fairly representative of the northwest suburbs of Mumbai. The centers cater to patients of diverse caste and creed, therefore lowering the risk of potential recruitment bias. After receiving written approval from both medical centers, all women attending the outpatient department for their routine antenatal check-up were invited to participate. The inclusion criteria were as follows: healthy women between 32 and 36 weeks of uncomplicated singleton pregnancy; no diagnosed medical disorders (e.g., gestational diabetes, preeclampsia); parity <3; and 20–35 years of age. A nurse along with the investigators provided information about the study to the attending pregnant women. Originally, 160 eligible participants showed interest. However, 68 affluent and 82 nonaffluent pregnant women (*N* = 150) arrived on the dates allocated for data collection, taking blood samples, recording sociodemographic characteristics, and 24-hour dietary recall.

### 2.2. Data Collection

Trained investigators via personal interview collected background information of the participants which included present age (years) and parity. Weight (kg) was noted from the medical records, which was measured on standardized digital weighing scales (Dr. Gene digital bathroom weighing scales, model no. MS8270) available at both medical centers, that is, private nursing home and government hospital. At each medical center, a trained nursing staff recorded the participants' weight. Heights of the participants were not recorded as they may not reflect their true height due to postural changes observed in the last trimester of pregnancy [12]. Prepregnancy heights and weights were not available in the medical records. The participants' economic status was indicated by the recruitment site: the private, fee-paying nursing home (affluent) versus the governmental, non-fee-paying hospital (nonaffluent). Neonatal anthropometric measurements were taken immediately after birth, namely, gestational age and birth weight. Gestational age was recorded as the mothers' date of delivery which was written by the obstetrician and computed as term delivery (≥37 weeks) versus preterm delivery (<37 weeks). Routinely well calibrated scales (Seca Digital Baby Scale Model no. 334), one at each recruitment site, were used to record infant's birth weight that measures weight to within 5 g. The measurements were taken by a trained nursing staff, one at each recruitment site, under the supervision of the field investigators, and categorized as healthy birth weight (≥2500 g) versus low birth weight (<2500 g).

### 2.3. Dietary Assessment

A 24-hour dietary recall was taken by trained investigators. Standardized food models, measuring cups, and spoons were used to record accurate estimates of portion sizes. For each recipe, the volume (mL), amount measured as number and grams, dimension, that is, thickness and diameter, and method of preparation were recorded for the raw ingredients and cooked yields as appropriate. The nutritive values provided by the National Institute of Nutrition, Hyderabad, India, were used to manually calculate the nutrient intakes from raw weights [13]. Intakes of calcium (mg), phytate (mg), phytate: calcium ratio (mmol/day phytate intake divided by mmol/day calcium intake), and phosphorus (mg) were calculated.

### 2.4. Calcium and Vitamin D Supplements

All pregnant women upon the advice of their obstetricians were consuming single calcium (500 mg)-vitamin D (250 IU or 6.25 *μ*g) supplement daily from the second semester onwards.

### 2.5. Sun Exposure

Sun exposure was calculated as an index, that is, hours/week the pregnant women spent outdoors in daylight multiplied by the percent body surface area exposed to sunlight. The type of clothing worn reflected the body surface area exposed. According to the rule of nines [14], the head and neck sun skin exposure accounts as 9%, each arm as 9%, each leg as 18%, and the front and back torso as 18% each. In our study, 9% (*n* = 13) women were veiled, exposing only the face and neck to sunlight (9% body surface area). “Veiled” in this study is not reflective of the “burkha” attire. It represents women covering their head and arms with their “*saree*” or “*duppatta*”/scarf worn with the “*salwar kameez.*” The remaining (*n* = 137, 91%) wore “*salwar kameez*” or “*saree,*” exposing their head, neck, and arms (27% body surface area). In addition, the time of the day to sun exposure in 24-hour format was also recorded.

### 2.6. Blood Analysis

The serum 25(OH)D levels were examined, which is the major circulating form with a half-life of 2-3 weeks, thus proposed to be the most reliable indicator of vitamin D adequacy [10] as it reflects both cutaneous synthesis and absorption from dietary sources [10]. Serum 25(OH)D was measured using the ARCHITECT 25-OH vitamin D chemiluminescent microparticle immunoassay (Abbott Diagnostics, Wiesbaden, Germany), which has shown to be comparable to the DiaSorin Liaison and DiaSorin radioimmunoassays [15]. Fasting blood (five mL) was collected by a trained phlebotomist from the antecubital vein in single vacutainer tubes (bar-coded gel tubes). The tubes were kept at room temperature for 30–60 minutes. The blood was then centrifuged at 2000 rpm for 10 minutes; serum was separated and transferred to eppendorfs tubes and immediately transferred to the laboratory for analysis. The cutoffs used to define vitamin D deficiency and insufficiency in pregnancy and the general populations are the same [10]. The Institute of Medicine has suggested 25(OH)D deficiency as levels <20.0 ng/mL (<50.0 nmol/L), 25(OH)D insufficiency as concentrations between 20.0 and 29.9 ng/mL (50.0–74.9 nmol/L), and adequate 25(OH)D levels as ≥30.0 ng/mL (≥75.0 nmol/L) [10].

### 2.7. Statistical Analysis

Logarithmic transformations were conducted to normalize the distribution of 25(OH)D (ng/mL) levels, dietary calcium (mg/d), phytate: calcium ratio, and the sun exposure index (percent body surface area exposed to sunlight × hours of sun exposure/week). Geometric means and 95% confidence intervals were reported for the log transformed variables and means ± SD were reported for variables normally distributed. Firstly, characteristics of the participants, namely, age (years), current weight (kg), parity (first pregnancy versus more than one pregnancy), sun exposure index, time of the day of sun exposure (24 hours), dietary calcium (mg/d), phytate : calcium ratio, dietary phosphorus (mg/d), and 25(OH)D status (ng/mL), were described according to their economic indicator, that is, affluent (private nursing home) versus nonaffluent (government hospital) using ANOVA or Pearson's chi-squared tests as appropriate for the continuous and categorical variables. Secondly, the prevalence of vitamin D inadequacy, defined according to the suggested cutoffs by the Institute of Medicine [10], was examined according to the participants' characteristics using ANOVA or Pearson's chi-squared tests as appropriate. Next, to study the association between participants' characteristics and 25(OH)D status (ng/mL), bivariate tests were conducted using ANOVA or Pearson's correlations as appropriate. The participants' characteristics with *P* values ≤ 0.20 were examined by multivariable linear regression to observe if the association with 25(OH)D remained robust. Lastly, the bivariate association between 25(OH)D status (ng/mL) and gestational age (term delivery: ≥37 weeks versus preterm delivery: <37 weeks) and birth weight (healthy birth weight: ≥2500 g versus low birth weight: <2500 g) were examined using ANOVA. Associations significant at *P* ≤ 0.20 were further examined by logistic regression, controlling for participants' characteristics. Checks for multicollinearity were performed by computing variance inflation factors which as recommended were below 10 for all variables [16]. There were no multivariate outliers and influential data points as all cases had Mahalanobis values below 25 and Cook's D values below one and were included in final analyses [16]. Significance for the regression analysis was set at *P* < 0.05. All analyses were conducted using SPSS version 21 (SPSS Inc., Chicago, USA).

## 3. Results

The overall mean age of the women was 26.8 ± 4.1 years and the mean weight was 62.4 ± 7.8 kg. The participants geometric mean serum 25(OH)D level was 10.6 ng/mL (95% CI: 10.0, 11.3 ng/mL) and their geometric mean sun exposure index was 47.5 (95% CI: 42.1, 53.7). The mean time of the day of sun exposure was 14:00 ± 2:00 hours. The women had geometric mean dietary calcium (315.3, 95% CI: 297.2, 334.5 mg/d), geometric mean phytate: calcium ratio (1.4, 95% CI: 1.3, 1.5), and mean phosphorus (590.4 ± 98.8 mg/d) intakes, respectively. The mean birth weight of the infants was 2.7 ± 0.4 kg and the mean gestational age of the infants was 35.5 ± 1.4 weeks.  Table 1 describes participants' characteristics according to their economic indicator, that is, affluent (private nursing home) versus nonaffluent (government hospital).

The prevalence of serum 25(OH)D adequacy by maternal characteristics is reported in Table 2. None of the pregnant women had adequate vitamin D concentrations (≥30.0 ng/mL). The majority of women (*n* = 141, 94%) were deficient in vitamin D (<20.0 ng/mL). In addition, mothers whose serum 25(OH)D levels were <20.0 ng/mL had significantly lower mean sun exposure index than women with vitamin D insufficiency (serum 25(OH)D between 20.0 and 29.9 ng/mL);  *F*(1,148) = 13.9,   *P* < 0.001.

**Figure 1:**
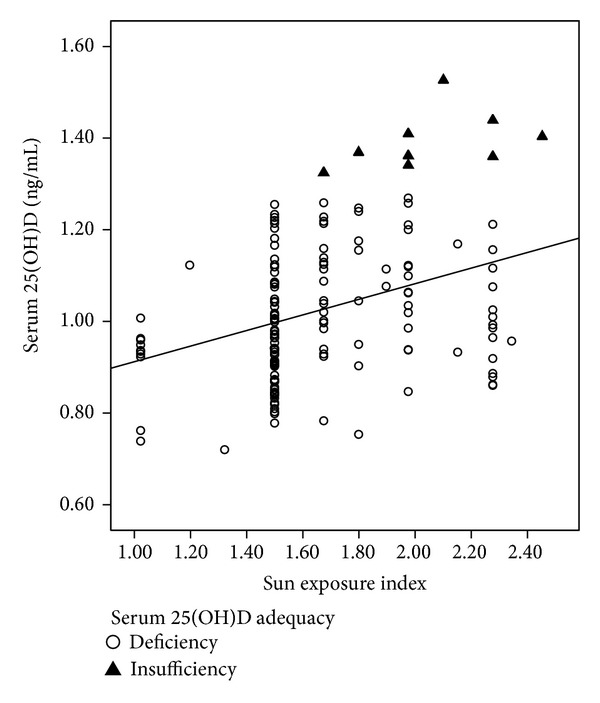
Association between the sun exposure index and serum 25(OH)D (ng/mL). None of the pregnant women had adequate vitamin D concentrations (≥30.0 ng/mL).

Table 3 reports association between maternal characteristics and serum 25(OH)D concentrations. At the bivariate level, maternal age, weight, phytate: calcium ratio, phosphorus intake, sun exposure index, time of the day of sun exposure, and economic indicator were associated with 25(OH)D status at *P* ≤ 0.20. The association between the selected independent variables and serum 25(OH)D concentrations was further examined using multivariable linear regression (Table 4). Results showed that the pregnant women recruited from the government hospital (nonaffluent class) were more likely to have poor vitamin D status in comparison to women from the private nursing home (affluent class) (*β* = −0.20; *P* = 0.03). Higher sun exposure index was associated with higher vitamin D status (*β* = 0.31; *P* < 0.001). In total, all variables explained 19% (*R* ^2^ = 0.19, adjusted* R* ^2^ = 0.16, *F*(6,143) = 5.6,   *P* < 0.001) of the variance in the serum 25(OH) vitamin D status of the pregnant women. The positive association between the sun exposure index and vitamin D status is also represented in Figure 1.

The association between serum 25(OH)D levels (ng/mL) and gestational age and birth weight were also examined. No association was observed between maternal vitamin D status and infants born at term (≥37 weeks, *n* = 41, geometric mean: 10.8, 95% CI: 9.7, 12.0) or preterm infants (<37 weeks, *n* = 109, geometric mean: 10.6, 95% CI: 9.9, 11.4); *P* = 0.79. At the bivariate level, mothers of infants with lower birth weight (<2500 g, *n* = 48, geometric mean: 9.6, 95% CI: 8.8, 10.6) had lower serum 25(OH)D concentrations in comparison to mothers of infants with healthy birth weight (≥2500 g, *n* = 102, geometric mean: 11.2, 95% CI: 10.4, 12.0); *F*(1,148) = 5.4,   *P* = 0.02. This association was further examined using logistic regression. The overall model examining the association between vitamin D status and infant birth weight was significant (chi-square = 26.9, *P* = 0.001, and Nagelkerke* R* ^2^ = 0.23). However, the association between maternal vitamin D status and infants birth weight became nonsignificant (OR: 0.17, CI: 0.01, 2.66, *P* = 0.21) after controlling for covariates (pregnant women's age, weight, parity, economic indicator, phytate: calcium ratio, dietary calcium, dietary phosphorus, sun exposure index, and time of day of sun exposure).

## 4. Discussion

This study principally examined vitamin D adequacy in pregnant women and investigated the relationship between lifestyle characteristics (sun exposure index, diet, and economic indicators) and serum vitamin D. The secondary aim was to study the association of serum 25(OH)D with birth weight and the gestational age. The principal finding highlighted that all pregnant women had vitamin D inadequacy. Secondly, the sun exposure index (percent body surface area exposed to sunlight × hours of sun exposure/week) and the economic indicator (affluent: private nursing home versus nonaffluent: government hospital) were significantly associated with the serum 25(OH)D levels after adjusting for potential confounding variables. Lastly, the secondary findings showed only a bivariate association between maternal 25(OH)D levels and birth weight. No relationship was observed between 25(OH)D concentrations and gestational age.

An important finding of our study was that all pregnant women irrespective of their economic class had vitamin D inadequacy. The prevalence rate observed was higher in comparison to reports in the existing literature. Previous reports indicated that 67%–97% of Indian nonaffluent pregnant women [7, 17–19] and 85% of affluent pregnant women [3] had vitamin D deficiency (<20.0 ng/mL) and/or insufficiency (20.1–29.9 ng/mL). The median serum 25(OH)D level observed in the present study for affluent pregnant women was only slightly higher (11.0 versus 10.4 ng/mL) than the value reported by Agarwal and Arya for a comparable group [3]. The mean/median (9.8/9.3 ng/mL) values observed for serum 25(OH)D for nonaffluent pregnant women in the present study were lower in comparison to most of the previous researches (median: 14.0–15.1 ng/mL) [7, 17, 18] except for the values (mean: 9.2–8.8 ng/mL) reported by Goswami et al. [2] and Marwaha et al. [19]. Similar to the present study, all previous researchers [2, 3, 7, 17, 18] have conducted a cross-sectional analysis on pregnant Indian women in the third trimester, with the number of subjects varying from as low as 20 to 541, and examined their serum 25(OH)D levels. Only, Marwaha et al. [19] recruited women in all three trimesters. However, true comparison between studies is difficult as the present study differed from previous researches on aspects such as vitamin D assays and period (gestational week) of collection, recruitment procedures, measures used to record the sun exposure, and seasonal and geographic variations. For example, several of the studies were conducted in North India, for example, Delhi [2, 3, 7, 19], and two studies were reported from South India (e.g., Mysore) [17, 18], whereas the present study was conducted in Mumbai City.

The results highlighted that the sun exposure index was positively associated with serum 25(OH)D concentrations. The sun exposure index remained a significant and a considerable (effect size: *β* = 0.31) independent variable even after controlling for covariates. Regrettably, the results also indicate that the sun exposure index might not be sufficient as all pregnant women in the study had serum vitamin D inadequacy. The available literature indicates that urban Indian predominantly nonaffluent pregnant women were exposed to sunlight for an average of 0.2 (~9 minutes) to 5.1 hours/day [2, 7, 19]. In comparison, pregnant women's exposure to sunlight in the present study was towards the lower end (0.3 hours/day ~21 minutes) of this range. Furthermore, a higher average sun exposure index (percent body surface area exposed to sunlight × hours of sun exposure/day) has been reported among rural pregnant Indian women in the literature [6] compared to our study according to the same definition (35.4 versus 9.2). This difference may partly be indicative of the discrepancies between the urban (e.g., housewives, office employees) and rural (e.g., housewives, but involved in outdoor agricultural activities) way of life [1]. Not only the duration of exposure to sunlight, but also the body surface area exposed is important for adequate vitamin D synthesis. In our study, we observed that the pregnant women exposed 9% (veiled)-27% (nonveiled) of their body surface area to sunlight. This was comparable with the report by Goswami et al. [20] and with those from other countries reporting vitamin D deficiency in Saudi (25%) and Israeli (37%) middle-eastern pregnant women wearing conservative clothing [8]. Therefore, the findings emphasize that, in a city such as Mumbai which experiences sunshine predominantly all- round the year (latitude: 18° 55′ N, longitude: 72° 50′ E, Zenith angle of 87.9° in peak summer and 47.5° in peak winter) [21], the limited duration of sun exposure and the modest traditional clothing norms such as the “*salwar kameez*” and “*saree*” may limit the synthesis and the adequacy of previtamin D and therefore serum 25(OH)D levels. Although the study did not record the skin type of the pregnant Indian women, defined according to the level of melanin, the majority of the indigenous Indian skin types (IV to V) may require two to three times longer sun exposure (0.75–1.5 h versus 0.25–0.5 h) than the lighter skinned Caucasians (types I, II, and III) to synthesize the same level of vitamin D [10]. These approximations are based on considerable skin exposure (face, full arms, and legs), which may be covered by the traditional Indian attire.

The economic indicator was significantly associated with the vitamin D status after adjusting for confounding variables. Results showed that nonaffluent pregnant women had lower serum 25(OH)D concentrations than their affluent counterparts. There are few reports in the literature wherein economic indicators of pregnant Indian women have been examined in relation to their vitamin D status. This finding may partly be explained by the reported observations that nonaffluent pregnant Indian women in our study had significantly (*P* < 0.001) lower dietary calcium and higher phytate to calcium ratio. In addition, nonaffluent women may have a lower sun exposure index, a trend that approached significance (*P* = 0.06).

Dietary indicators (dietary calcium, dietary phosphorus, and phytate: calcium ratio) were not significantly associated with maternal serum vitamin D status. Dietary factors may only provide a small contribution, but they may be crucial for Indians because the dermal synthesis of vitamin D may be low due to culturally specific reasons already discussed [2, 7]. Results showed that the mean calcium intake (315.3 mg/d) of pregnant women met only 31.5% (1000 mg/d) of the total recommended intake by the Indian Council of Medical Research [22], which is consistent with studies on pregnant Indian women [19]. This is of concern as low dietary calcium converts 25(OH)D into polar metabolites in the hepatic cells, which is associated with secondary 25(OH)D deficiency [9].

Lastly, secondary findings observed no significant relationship between maternal serum 25(OH)D levels and infant birth weight after controlling for covariates. Previous research has reported mixed findings. Several studies have shown no association [23–26], whereas one cross-sectional study (*N* = 461) from Australia reported a significant association between the infants' low birth weight and vitamin D deficiency status of their mothers (<25.0 nmol/L) [8]. In contrast, even higher birth weights are reported among infants with vitamin D deficient mothers (<37.5 nmol/L) [27]. Therefore, longitudinal research is required to systematically examine the relationship between maternal vitamin D status and birth weight.

The study findings are strengthened as validated procedures were used to measure serum vitamin D and record the dietary intake data. However, findings should be interpreted keeping in consideration the limitations. The study was cross-sectional in nature; relationships may vary over time, therefore emphasizing the need for longitudinal research. The convenience and small sample size limit the observations to affluent and nonaffluent pregnant Indian women attending the private nursing home and government hospital in northwest suburbs of Mumbai. Confounding variables such as seasonal variations, skin pigmentation, vitamin D intake, serum calcium, serum phosphorus, parathyroid levels, and prepregnancy measured height and weight were not recorded. This was due to pragmatic issues such as limited funds, time, trained technicians, and constraints at the site of data collection, as the study was conducted in a very busy and overcrowded private nursing home and in a government hospital.

## 5. Conclusion

In conclusion, all pregnant affluent and nonaffluent Indian women had vitamin D inadequacy and the study showed that the sun exposure index was a significant factor associated with maternal serum 25(OH)D levels. However, recommending pregnant women to increase the duration of sun exposure may not always be practical, and it would be culturally unacceptable to suggest a decrease in the area of skin covered by clothing. Therefore, the study proposes alternative take-home messages that might be better suited for the affluent and nonaffluent pregnant Indian women. All pregnant women in the study consumed a calcium-vitamin D supplement daily. Most of the prenatal multivitamin and mineral supplements prescribed in the US contain 400 IU/day vitamin D [10]. Thus, the women in the present study consumed 62.5% (250 IU/day) of the suggested supplemental dose of vitamin D. In India, mandatory vitamin D supplementation is not part of the antenatal care program, but it could be well-suited in light of the universal vitamin D deficiency noted in the study. Another approach could be vitamin D fortification of staple foods to meet the recommended daily allowance of vitamin D (600 IU/day) for pregnant women set by the Institute of Medicine in the US [10] (vitamin D recommendations for Indians are not yet developed by the ICMR). In India, vitamin D fortified foods and dietary fats are not available and the natural dietary sources of vitamin D may not be affordable to all. Therefore, staple food such as wheat could be fortified, which is consumed by all ages, religious backgrounds, and urban and rural sectors and which is easily accessible and affordable by all economic strata.

## Figures and Tables

**Table 1:** Characteristics of the affluent and nonaffluent mothers (*N* = 150).

Maternal characteristics	Affluent: private nursing home (*n* = 68)	Nonaffluent: government hospital (*n* = 82)	
Mean ± SD or (95% CI)^4^			*P* value

Age (years)	27.8 ± 3.9	26.0 ± 3.9	**0.008**
Weight (kg)	61.9 ± 7.9	62.7 ± 7.6	0.58
Phytate : calcium^1,2^	1.3 (1.2, 1.4)	1.5 (1.4, 1.6)	**<0.001**
Calcium (mg/d)^1^	353.9 (323.4, 387.3)	286.4 (266.0, 308.4)	**<0.001**
Phosphorus (mg/d)	587.6 ± 99.2	592.8 ± 98.9	0.75
Sun exposure index^1,3^	53.4 (44.2, 64.6)	43.1 (36.8, 50.5)	0.06
Time of day (24 hours)	13:00 ± 2:00	14:00 ± 2:00	0.42
25(OH)D (ng/mL)^1^	11.8 (10.8, 12.9)	9.8 (9.1, 10.6)	**0.002**

% (*n*)^5^			*P* value

Parity			
First pregnancy	46.6 (55)	53.4 (63)	0.55
>1 pregnancy	40.6 (13)	59.4 (19)	

^1^Log transformed. Geometric means and 95% confidence intervals reported.

^2^Phytate : calcium ratio = mmol/day phytate intake/mmol/day calcium intake.

^3^% body surface area exposed to sunlight × hours of sun exposure/week. Higher values indicate greater exposure to sunlight.

^4^ANOVA.

^5^Pearson's chi-squared tests.

Time of day (24 hours) refers to the time of the day to sun exposure in 24-hour format.

**Table 2:** Maternal characteristics by 25(OH)D adequacy (*N* = 150).

Maternal characteristics	25(OH)D deficiency <20.0 ng/mL (*n* = 141)	25(OH)D insufficiency 20.0–29.9 ng/mL (*n* = 9)	
Mean ± SD or (95% CI)^4^			*P* value

Age (years)	26.7 ± 4.1	27.8 ± 2.1	0.46
Weight (kg)	62.5 ± 7.6	59.8 ± 8.8	0.31
Phytate : calcium^1,2^	1.4 (1.3, 1.8)	1.3 (1.2, 1.4)	0.11
Calcium (mg/d)^1^	312.8 (294.0, 332.7)	357.8 (284.4, 449.9)	0.29
Phosphorus (mg/d)	590.9 ± 97.9	581.9 ± 117.2	0.79
Sun exposure index^1,3^	44.9 (39.8, 50.8)	113.8 (73.5, 176.2)	**<0.001**
Time of day (24 hours)	14:00 ± 2:00	13:00 ± 3:00	0.30

% (*n*)^5^			*P* value

Hospital			
Private	91.2 (62)	8.8 (6)	0.19
Government	96.3 (79)	3.7 (3)	
Parity			
First pregnancy	93.2 (110)	6.8 (8)	0.44
>1 pregnancy	96.9 (31)	3.1 (1)	

^1^Log transformed variables. Geometric means and 95% confidence intervals reported.

^2^Phytate : calcium ratio = mmol/day phytate intake/mmol/day calcium intake.

^3^% body surface area exposed to sunlight × hours of sun exposure/week. Higher values indicate greater exposure to sunlight.

^4^ANOVA.

^5^Pearson's chi-squared tests.

Time of day (24 hours) refers to the time of the day to sun exposure in 24-hour format.

**Table 3:** Bivariate associations between maternal characteristics and 25(OH)D status (ng/mL) (*N* = 150).

Maternal characteristics	*r* value^4^	*P* value
Age (years)	0.17	**0.04**
Weight (kg)	−0.11	**0.18**
Phytate : calcium^1,2^	−0.11	**0.18**
Calcium (mg/d)^1^	0.09	0.25
Phosphorus (mg/d)	0.10	**0.20**
Sun exposure index^1,3^	0.35	**<0.001**
Time of day (24 hours)	−0.11	**0.18**

	Mean (95% CI)^5^	*P* value

Hospital		
Private nursing home (*n* = 68)	11.8 (10.8, 12.9)	**0.002**
Government (*n* = 82)	9.8 (9.1, 10.6)	
Parity		
First pregnancy (*n* = 118)	10.6 (9.9, 11.3)	0.71
>1 pregnancy (*n* = 32)	10.9 (9.5, 12.5)	

*P* ≤ 0.20 were considered for further multivariable linear regression analysis.

^1^Log transformed variables. Geometric means and 95% confidence intervals reported.

^2^Phytate : calcium ratio = mmol/day phytate intake/mmol/day calcium intake.

^3^% body surface area exposed to sunlight × hours of sun exposure/week. Higher values indicate greater exposure to sunlight.

^5^ANOVA.

^4^Pearson's correlations.

Time of day (24 hours) refers to the time of the day to sun exposure in 24-hour format.

**Table 4:** Simultaneous linear regression between specific maternal characteristics and 25(OH)D status (ng/mL) (*N* = 150).

Maternal characteristics	*β* value	*P* value
Age (years)	0.12	0.13
Weight (kg)	−0.01	0.89
Phytate : calcium^1,2^	−0.03	0.72
Phosphorus (mg/d)	0.11	0.16
Hospital (government) Referent group: private nursing home	−0.20	**0.03**
Sun exposure index^1,3^	0.31	**<0.001**
Time of day (24 hours)	−0.08	0.30

Dependent variable: 25(OH)D status (ng/mL).

*R* ^2^ = 0.19, adjusted *R* ^2^ = 0.16, *F* (7, 142) = 5.0, and *P* < 0.001.

^1^Log transformed variables.

^2^Phytate : calcium ratio = mmol/day phytate intake/mmol/day calcium intake.

^3^% body surface area exposed to sunlight × hours of sun exposure/week. Higher values indicate greater exposure to sunlight.

Time of day (24 hours) refers to the time of the day to sun exposure in 24-hour format.
